# Chronic inflammation on gingiva-derived mesenchymal stem cells

**DOI:** 10.6026/97320630019138

**Published:** 2023-01-31

**Authors:** Anant Ragav Sharma, Rakesh Kumar Jaiswal, Swapnali Shinde Kamble, Mahesh Ghadage, Madhura Pawar, Hiroj Bagde, Ramanpal Singh Makkad

**Affiliations:** 1 Department of Periodontics, Pacific Dental College, Debari, Udaipur, Rajasthan, India; 2 Department of Dentistry, Late Shree Lakhiram Agrawal Memorial Government Medical College Raigarh Chhattisgarh Pin 496001, India; 3 Department of Preventive and Pediatric Dentistry, Child Dental Home, Borivali (W), India; 4 Department of Prosthodontics and Crown and Bridge.Bharati Vidyapeeth (Deemed to be University) Pune Dental College and Hospital, Navi Mumbai, India; 5 Department of Pediatric and Preventive Dentistry, Dr D Y Patil Dental College and Hospital, Dr D Y Patil Vidyapeeth Pimpri, Pune, Maharashtra, India; 6 Department of Periodontology, Rama Dental College, Hospital and Research Centre, Kanpur; 7 Department of Oral Medicine and Radiology, New Horizon Dental College and Research Institute, Bilaspur, Chhattisgarh, India

**Keywords:** Chronic inflammation, gingiva-derived mesenchymal stem cells, influence

## Abstract

The impact of inflammatory response on the biological characteristics of GMSCs has been the subject of studies, with conflicting findings. In order to more fully understand the effects of the localized inflammatory environment, the current study assessed the intensity and differentiating capacity of GMSCs derived from healthy periodontal tissues (H-GMSC) and GMSC derived from inflamed periodontal tissues (I-GMSC) tissues. Cells from every well were taken out and counted using a hemocytometer every three days for a total of 12 days. The findings of the cell proliferation assay, which involved quantifying the cells with the help of a hemocytometer at 0th day, 3rd day, 6th day, and 9th day, are shown. On day nine of culture, there was a considerable (P = 0.02) variation in the rate of multiplication between GMSCs from healthy gingival tissues and GMSCs from gingival tissues having inflammation. Additionally, I-GMSCs had a higher cell concentration on day twelve than that of H-GMSCs. However, there was no significant variance in PDT values comparing GMSCs from healthy gingival tissues and GMSCs from gingival tissues having inflammation (P > 0.05). The mean PDT findings of 66.7 h and 53.4 h have been documented for Healthy-GMSCs and Inflamed-GMSCs, respectively. In addition, compared to GMSCs from healthy gingival tissues, GMSCs from inflammatory tissues had decreased osteogenesis and increased adipogenic potential. To evaluate the efficacy of GMSCs derived from patients suffering periodontitis utilising human models for cell-based treatments, additional study is necessary.

## Background:

A persistent inflammatory condition called periodontitis causes the tissues that support teeth to gradually deteriorate. The main objective of periodontal therapy is to protect the tooth structure in function and appearance of the tooth structure in function and appearance is the main objective of periodontal therapy. By reducing inflammation and repairing missing tooth-supporting tissues, this is accomplished. Due to the inability to manage an abnormal inflammation and the paucity of progenitor cells at the location of the defect, existing medical approaches to the rejuvenation of periodontium have met with various degrees of success. [1] Alternative tactics utilising biologic mediators could therefore get around these problems. [2] Cell-based therapy has become more popular among the various methods for encouraging healing in a predictable way. With their capacity for auto regeneration, multi potential differentiation, as well as immune modulatory features, the mesenchymal stem cells (MSCs) is important tissue regeneration sources. [3] There is scientific proof that MSCs are present in dental tissues. [4] Gingiva-derived mesenchymal stem cells (GMSCs) first were isolated and characterized in 2009 [5], and it was found that these cells were capable of multi lineage transformation, self-regeneration, and identical clone formation. [5,6,7] GMSCs have demonstrated stem cell pluripotent traits and are mostly produced from cranium neural crest cells. [8,9] Given its rapid ex vivo proliferation, simplicity of isolation, immuno regulatory qualities both outside and inside body [5, 6,7,8,9,10-11], the connective tissue of gingiva has been suggested as a potential alternative option of MSCs for regeneration of periodontal tissues. [8,9,10, 11,12,13] The origin of GMSCs in patients receiving therapy for periodontitis is most probably the inflammatory gingival tissue. MSC-like cells have been discovered in disease ridden dental tissue in earlier studies. [14-15,1616] MSCs from inflammatory pulp tissue and periodontal ligament tissue revealed defective immune modulatory capabilities. Nonetheless, they preserved their regeneration potential. Studies examining how inflammation affects GMSCs found fewer alterations caused by inflammation. [7,8,9,10,11,12, 13,14,15,16,17, 18] However, it was shown that inflammation sped up the rate at which their population doubled (PDT). [14] In addition, the inflammatory environment caused the GMSCs to specialize more towards a profibrotic pattern while still maintaining a high reproductive activity. [15,16,17,18,19] The impact of inflammatory response on the biological characteristics of GMSCs has been the subject of studies, with conflicting findings. In order to more fully understand the effects of the localized inflammatory environment, the current study assessed the intensity and differentiating capacity of GMSCs derived from healthy periodontal tissues (H-GMSC) and GMSC derived from inflamed periodontal tissues (I-GMSC) tissues.

## Material and Methods:

When participants had routine extractions for orthodontic considerations, crown lengthening procedures, or during intentional third molar surgery, healthy tissues from gingiva were taken from them. Gingiva from inflammatory tissue was taken during an accessible periodontal flap surgical intervention from patients who had been diagnosed clinically with chronicperiodontitis. [14] Prior to collecting the tissues, the patients provided their informed consent. The tissues utilized in the study were those that would typically be thrown away after the surgery. For the purpose of creating cell lines with the least amount of heterogeneity, a maximum of ten tissue specimens from each category were collected. The partial enzyme digestion approach, which was used to separate GMSCs from healthy periodontal tissues and inflammatory periodontal tissues, was modified somewhat from the previously described protocol.[14] In a nutshell, the tissues were cut into 1-2 mm pieces after being rinsed three times with Dulbecco's phosphate-buffered solution (Gibco, USA). The remaining tissue was then placed in 0.1percentage type IV collagenase enzyme (Gibco) for duration of 1 hour, after which the enzyme-digested specimens were centrifuged for 5 minutes at 1000 rpm. Then, 4-5 tissue specimens were incubated for an overnight period at 37°C in a humid environment of 5% CO2 in the air with a minimum quantity of growth promoting medium consisting of 10 percent of fetal bovine serum ( termed as FBS, Gibco), 100 U/mL of penicillin, and 100 g/mL of streptomycin (Gibco). Cells were removed using a 0.1percent (weight/volume) trypsin-ethylenediaminetetraacetic acid mixture (Gibco) after the culture was maintained until the cells achieved 70%-80% proliferation. For additional analysis, cells were subsequently sub passaged three to five times, with each passage lasting twelve to fifteen days. With the help of a phase-contrast microscope, cells were examined for adhesion and morphology throughout the cultivation (Olympus, Japan). Each GMSC passage from Passage one (P1) to Passage five (P5) was used to calculate the proportion of viable cells. By utilising a hemo cytometer to stain cells with 0.4% trypan blue (Gibco), viability of cell was evaluated. Transparent cells were considered and counted as live cells, while those that stained blue were thought to be dead. By plating 5000 cells per well on a tissue culture plate having twelve wells (Thermo Scientific, USA) in three replications, the multiplication and PDT of GMSCs were examined. Cells from every well were taken out and counted using a hemocytometer every three days for a total of 12 days. Every three days, the culture media would be replaced. The formula used to determine PDT for GMSCs is PDT = t (log2)/(log Nt-log No), where t is the culture period and No and Nt represents the cell counts before and following seeding, respectively. The methods previously described were used, with a few minor adjustments, to evaluate the strength and differentiating capacity of GMSCs. [8]. By cultivating GMSCs on a plate consisting of twelve wells at a density of 50 cells per cm2 for 15 days, the ability to form colonies was tested. Once every three days, the media was replaced. For staining, cells were pretreated with 3.7 percentage paraformaldehyde solution (Sigma-Aldrich, USA), labeled for twenty minutes at room temperature with 1percent Giemsa stain (Sigma-Aldrich), and looked at under the microscope (Olympus).

## Statistical analysis:

The data collected in the form of mean with standard deviations. Intergroup difference was evaluated using the Kruskal-Wallis H test (non-parametric ANOVA.). Intra group groups, differences were compared using Mann Whitney U test. The confidence level was maintained at 95% with the result that a P-value below 0.05 indicated a statistically significant association. SPSS software version 22 (IBM, USA) was used for carrying out all statistical analyses.

## Results:

From 24 to 48 hours after the discharge of H-GMSCs as well as I-GMSCs from the tissue specimens began, this was seen. On the fifth day after the tissue was placed on the culture plate, the cells became plainly visible. Both GMSCs initially showed a heterogeneous combination of cells with subtly different morphologies. By day 10 day of culture, plastic adhered GMSCs exhibited a distinctive shape resembling fibroblasts. Both GMSCs had 80%–90% confluency and cells with a long, thin shape on day 15. At every examined passage, GMSCs from healthy gingival tissues and GMSCs from gingival tissues having inflammation demonstrated >95% vitality, and no significant variations (P > 0.05) in frequency and percentage were found between the isolates at various passages. The findings of the cell proliferation assay, which involved quantifying the cells with the help of a hemocytometer at 0th day, 3rd day, 6th day, and 9th day, are shown. On day nine of culture, there was a considerable (P = 0.02) variation in the rate of multiplication between GMSCs from healthy gingival tissues and GMSCs from gingival tissues having inflammation. Additionally, I-GMSCs had a higher cell concentration on day twelve than that of H-GMSCs. However, there was no significant variance in PDT values comparing GMSCs from healthy gingival tissues and GMSCs from gingival tissues having inflammation (P > 0.05). The mean PDT findings of 66.7 h and 53.4 h have been documented for Healthy-GMSCs and Inflamed-GMSCs, respectively. The GMSCs' capacity to form colonies was an indication of the abundance of mesenchymal clonogenic progenitors in tissue of gingiva. While GMSCs from inflamed gingiva displayed numerous, bigger colonies, GMSCs from healthy gingiva showed lesser, shorter colonies. Colony producing unit-fibroblast colonies were defined as collections of at least 50 cells. Outcomes of analysis using flow cytometry for GMSCs from healthy gingiva and GMSCs from inflamed gingiva are given. SSEA4 and Stro1 transcription varied in strength and was marginally less pronounced in both GMSCs. However, hematopoietic cell indicators like CD34 marker and CD45 marker, which showed extremely low expression in contrast to MSC-associated indicators like CD73 marker and CD90 marker. In I-GMSCs, CD73 and CD90 expression was higher, indicating a function in preserving stemness characteristics. Both GMSCs had minimal representation of CD105 marker, a hallmark for cells committed to a certain lineage. Outcomes of both GMSCs osteogenic capability are reported. The structure of GMSCs kept as controls remained fibroblastic for the entire 21-day culture period. Cell size decreased and became irregular after osteogenic induction, taking on a somewhat morepolygonal appearance. Later, von Kossa labeling in GMSCs from healthy gingiva and GMSCs from inflamed gingiva revealed the accumulation of mineralized clusters in the cultures. When compared to I-GMSCs, H-GMSCs had a somewhat stronger osteogenic capacity. The adipogenicity in GMSCs from healthy gingiva and GMSCs from inflamed gingiva data are shown. GMSCs from monolayer cultures fed with adipogenic stimulating media exhibited small vacuoles, as opposed to the paucity of these structures in the control samples. Comparing GMSCs from inflamed gingiva to GMSCs from healthy gingiva, a slight increase in adipogenic capability was seen on 21st day.

## Discussion:

GMSCs have more accessibility as compared to the neural stem cells based in tissues of periodontium, which possess an innate capacity to transform into diverse tissues required for development of structures that support tooth in human body. Therefore, GMSCs may be an alternate option for cell-based therapies and tissue regeneration. [20] The inflamed gingiva serves as the resource of GMSCs that can be used in cell-based intervention in individuals with widespread periodontitis. The capacity of these cells to maintain their MSC capabilities and tolerate infection in the context of an inflammatory milieu may have been acquired by repeated exposure to oral microbiota and mechanical activation during mastication. [9] However, there is conflicting evidence about how long-lasting inflammation affects GMSC potency. Because of the particular ecosystem in within which they are continually exposed, it has been theorized that MSCs produced from gingiva have characteristics that set them apart from MSCs from other dental tissues. [9] By partially digesting tissue of gingiva with 0.1 percentage type IV collagenase enzyme, which allowed for the imminent production of cells with plastic adhesion capacity, GMSCs were separated and characterised from healthy periodontal tissues and inflamed tissue of gingiva in the current investigation. For collecting cell suspension and creating primary cultures, prior investigations used collagenase enzyme and dispase enzyme in conjunction. [5,6,7,8,9, 10,11,12] By fifteen day of cell cultures, tissue of gingiva in semi digested condition employed in our research as an explant produced more GMSCs from healthy gingiva and GMSCs from inflamed gingiva with higher homogeneity. It is hypothesized that a reduced incubation time with a specific enzyme reduced its impact on GMSCs and increased the number of cells produced. The viability property, proliferation property, and doubling time property of cells as well as other growth kinetic characteristics are typically used to determine the capability of GMSCs to auto-renewal during in vitro condition. These findings offer important information for the potential use of GMSCs in treatment. GM SCs from both categories in this research demonstrated >95 percentage survivability from P1 phase to P5 phase. Additionally, during culture proliferation, these cells exhibited a lower PDT and were very proliferative. While the rate of proliferation and time taken to double the number of cells demonstrate capability for development of culture, the analysis of colony-forming pattern provides an indication regarding the quality of MSCs that are located inside the tissue. The quantity of adhering CFU-F did not significantly differ between the I-GMSCs and H-GMSCs, despite the latter showing more colonies and larger ones than the former. The creation of somewhat more colonies in I-GMSCs may be due to increased proliferation rate. Previous research has shown that GMSCs have a better capacity for clone formation than periodontal ligament stem cells. [17] As a result, it was hypothesized that inflammation might stimulate I-GMSCs. These results support earlier research that indicated inflammation had an induive influence on the capability of GMSCs to proliferate. [7,8,9, 10,11,12,13,14, 15,16,17] The diverse lineage commitments of MSCs, which may be influenced by their in vivo environment, make up their heterogeneous population. [9]. Both GMSCs from healthy gingiva and GMSCs from inflamed gingiva were found to have marginal magnitude for SSEA4 marker, Stro1 marker, and CD105 marker, sufficient magnitude for CD73 marker, and deficient magnitude for the different hematopoietic cell indicators like CD34 markers and CD45 markers after MSC surface markers were examined. The stromal ancestry of the both GMSCs in culture evaluated in our study was validated by the immuno phenotypic profiles. The CD73 and CD90 expression was very positive in GMSCs from healthy gingiva and GMSCs from inflamed gingiva. Elevated expression levels of these indicators are consistent with earlier findings. [5,6,7,8, 9,10,11,12,13, 14,15,16,17,18, 19,20,21] Stro1 may aid in the homing and angiogenesis of MSCs and has been linked to clonogenicity. It is still unknown if Stro1 expression and multipotency are related. An embryonic stem cell marker called SSEA4 indicates that MSCs are clonogenic and multi potent. [22] Furthermore, periodontally healthy gingival tissue had more Stro1- and SSEA4-positive MSCs than did diseased gingival tissue. [15] SSEA4 and Sto1 had a mild-to-low reactivity in our data, but there was no significant variance in their translation between the groups with healthy gingiva and those with inflamed gingiva. Stro1 expression may gradually decrease with culture, which could account for the low positive of Stro1 in present research. [2222] Our investigation found that whereas CD105 is weakly positive in healthy gingiva as well as inflamed gingival tissues, it is significantly positive in MSCs. Overall, the findings imply that inflammatory process did not have the same impact on expression of markers of stem cells as had been described earlier. [14,15, 16,1717] We found that I-GMSCs still possessed proliferative characteristics but had a lower osteogenic potential and a higher capacity for adipogenic differentiation. Similarly, MSCs from inflammatory periodontal ligament in earlier research shown a reduced ability for development of nodules of mineralization in relation to those derived from gingival tissue that were healthy. [23] It was postulated that excessive pro-inflammatory cytokine production during periodontitis may influence the process of osteoblastic delineation of MSCs through a variety of regulatory processes, including suppression of nuclear factor-kappa B biomarker by means of catenin signaling, which suppresses miR-21 biomarker , and/or RUNX2 biomarker inhibition. [24- 25] However, a recent study found that when GMSCs from the inflamed region were cultivated in cytokine-preconditioned medium, their osteogenic capacity was retained. [26]

## Conclusion:

The current study's findings confirmed that GMSCs can be retrieved from both healthy gingival tissue and gingival tissues having inflammation, and that the inflammatory condition may have an influence on the kinetics of growth of GMSCs and capacity for formation of colony. In addition, compared to GMSCs from healthy gingival tissues, GMSCs from inflammatory tissues had decreased osteogenesis and increased adipogenic potential. To evaluate the efficacy of GMSCs derived from patients suffering periodontitis utilising human models for cell-based treatments, additional study is necessary.

## Figures and Tables

**Table 1:** Data regarding the values of H-GMSC and I-GMSC at different time

	H-GMSC-1	H-GMSC-1	I-GMSC-1	I-GMSC-2	P value
P1	86.12±2.34	87.23±4.67	88.34±3.78	88.23±3.34	0.65
P2	85.23±1.46	86.45±3.64	87.25±4.67	87.56±4.56	0.56
P3	87.34±3.67	87.12±4.67	88.23±6.23	88.27±3.12	0.34
P4	86.45±4.56	86.34±3.32	87.43±5.76	88.45±2.12	0.68
P value	0.43	0.67	0.78	0.64	
